# Antifungal and Antivirulence Activities of Hydroalcoholic Extract and Fractions of *Platonia insignis* Leaves against Vaginal Isolates of *Candida* Species

**DOI:** 10.3390/pathogens9020084

**Published:** 2020-01-28

**Authors:** Anderson França da Silva, Cláudia Quintino da Rocha, Luís Cláudio Nascimento da Silva, Alexsander Rodrigues Carvalho Júnior, Iven Neylla Farias Vale Mendes, Andrea Borges de Araruna, Elizangela Pestana Motta, Rayssa de Sousa Silva, Carmem Duarte Lima Campos, Josivan Regis Farias, Aluísio da Silva Oliveira, Douglas Henrique dos Santos Silva, Flávia Raquel F Nascimento, Rosane Nassar Meireles Guerra, Cristina Andrade Monteiro

**Affiliations:** 1 Laboratório de Imunofisiologia, Programa de Doutorado em Biotecnologia—RENORBIO, Universidade Federal do Maranhão, 65085-580 São Luís, Ma, Brazil; andersonsilva.ppgcs@ufma.br (A.F.d.S.); andreaborges19@hotmail.com (A.B.d.A.); elifarmabr@gmail.com (E.P.M.); regis.farias95@gmail.com (J.R.F.); as.olivira@ufma.br (A.d.S.O.); flavia.nascimento@ufma.br (F.R.F.N.); 2 Laboratório de Química de Produtos Naturais, Centro de Ciências Exatas e Tecnológicas, Universidade Federal do Maranhão, 65085-580 São Luís, Ma, Brazil; rocha.claudia@ufma.com.br; 3 Laboratório de Imunologia das Doenças Infecciosas e Parasitárias, Programa de Mestrado em Biologia Microbiana, Universidade Ceuma, 65075-120 São Luís, Ma, Brazilaecarvalhojr@usp.br (A.R.C.J.); 4 Departamento de Ensino, Instituto Federal do Maranhão, 65485-000 Itapecuru-Mirim, Ma, Brazil; iven.mendes@ifma.edu.br; 5 Laboratório de Microbiologia Aplicada, Programa de Biologia Microbiana, Universidade Ceuma, 65075-120 São Luís, Ma, Brazil; ra.silva2706@gmail.com (R.d.S.S.); carmemdlcampos@gmail.com (C.D.L.C.); 6 Laboratório de Microbiologia Aplicada, Curso de Especialização em Microbiologia Clínica, Universidade Ceuma, 65075-120 São Luís, Ma, Brazil; douglasbmdh@gmail.com; 7 Departamento de Biologia, Instituto Federal do Maranhão, 65030-005 São Luís, Ma, Brazil

**Keywords:** *Candida albicans*, *Candida glabrata*, vulvovaginal candidiasis, *Platonia insignis*, biofilm, antifungals

## Abstract

Vulvovaginal candidiasis is a common fungal infection in women. In this study, *Platonia insignis* hydroalcoholic extract (PiHE) and its fractions were evaluated for antifungal and antivirulence activities against vaginal *Candida* species. Dichloromethane (DCMF) and ethyl acetate fractions (EAF) obtained from PiHE effectively inhibited the pathogen. Electrospray ionization mass spectrometry was used for identifying the main compounds in extracts. Minimal inhibitory and fungicidal concentrations (MIC and MFC, respectively) were determined by a broth microdilution assay. Furthermore, we evaluated the effect of the extract and fractions on the virulence properties of *Candida albicans*, and their cytotoxicity effect was determined on RAW 264.7 cells. Compounds found in extracts were flavonoid glycosides, mainly derivatives of quercetin and myricetin. Extracts showed antifungal potential, with the lowest MIC value for EAF (1.3 mg/mL) and inhibited *Candida* adherence and biofilm formation. EAF disrupted 48 h biofilms with an inhibition rate of more than 90%. The extract and its fractions exhibited no cytotoxicity. The antifungal effects were attributed to the ability of these extracts to alter the mitochondrial membrane potential for the release of pro-apoptotic factors in the cytosol. In conclusion, our data suggest that PiHE and EAF could act as novel candidates for the development of new therapeutic treatments against fungal infections.

## 1. Introduction

*Candida albicans* is considered the main etiologic agent of vulvovaginal candidiasis (VVC) and is responsible for 80%–85% of cases, followed by *Candida glabrata*, accounting for 10%–20% of cases [1]. VVC is the most prevalent acute human fungal infection, affecting 50%–75% of women of a childbearing age once in their lifetime, with a 5%–8% chance of developing the severe chronic form, known as recurrent vulvovaginal candidiasis (RVVC) [2]. A recent study estimated that annually, at least 138 million women worldwide are affected by RVVC [3,4].

Risk factors for VVC include pregnancy, oral contraceptives, diabetes mellitus, treatment with broad-spectrum antibiotics, steroids, and immunosuppressive therapies [5]. Virulence factors expressed by *Candida* spp. are also important for the development of infection, such as the ability to form biofilms [6]. About 80% of microorganisms live in biofilms [7] and sessile cells within *Candida* biofilms are resistant to antifungal agents [8].

Currently, the most commonly used antifungal agents for treating VVC are azoles. The treatment of fungal infections has become challenging because of the rapid development of antifungal resistance due to the over-prescription of medications and prophylactic treatments. Adverse effects presented by antifungals, such as cytotoxicity, gastrointestinal disorders, and hepatotoxicity, contribute to the phenomenon [8,9]. Therefore, it is necessary to develop new antifungal agents against *Candida* spp. to improve the clinical management of fungal diseases.

In Brazil, secondary plant metabolites have been traditionally used for treating fungal diseases due to their antifungal activity. Extracts obtained from plant species native to Brazil that have antifungal activity and exhibit a low cytotoxicity may act as alternatives to the conventional drugs used for the treatment of VVC [8,10].

*Platonia insignis* belongs to the Clusiaceae family and is popularly known as “bacurizeiro”. Native to the Brazilian Amazon forests, *P. insignis* is a fruit-bearing and woody plant with a dense and diverse distribution throughout the country [11]. Traditionally, it has been used to treat various ailments related to the digestive tract and rapid wound healing, and was also reported to have diuretic and antitumor properties [12,13].

Previous natural product studies have focused on the characterization of fats extracted from seeds used in folk medicine to treat diarrhea, skin problems, earaches, rheumatism, arthritis, and scarring [12,13,14]. There have been some reports investigating the effects of *P. insignis* seed compounds as antioxidants, anti-inflammatory agents, cytotoxic agents, and leishmanicidal agents. These were also investigated for their use in treating Alzheimer’s and Parkinson’s diseases. However, *P. insignis* leaf extracts have not been well-characterized in terms of their use in treating *Candida* infections. In this study, we analyzed the phytochemical composition and investigated the in vitro antifungal and antivirulence potential of a hydroethanolic extract of leaves of *P. insignis* (PiHE), and the dichloromethane (DCMF) and ethyl acetate fractions (EAF) against *Candida* spp. isolated from vaginal secretions. Furthermore, we verified the cytotoxicity and the effect of these extracts on fungal cells. We hope that leaf extracts of *P. insignis* can be used in developing effective therapeutic agents against *Candida* infections.

## 2. Results

### 2.1. Phytochemical Characterization

**Figure 1:**
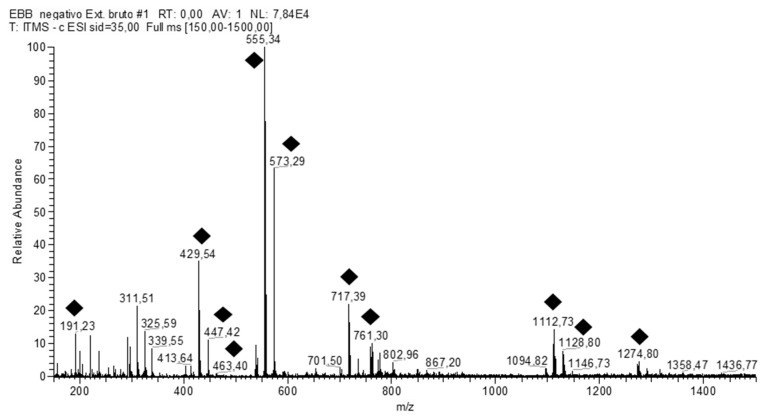
Representative direct flow injection analysis FIA-ESI-IT-MS fingerprint spectra obtained in negative ion mode of the 70% EtOH extract from the leaves of *Platonia insignis.* (♦) represents various fragmented constituents.

The qualitative profiles of PiHE were analyzed by FIA-ESI-IT-MS/MS^n^ and HPLC-PAD-ESI-MS techniques and this resulted in the unambiguous identification of 13 compounds. The full scan spectrum of the ions obtained is shown in Figure 1, and the fragmentation data recorded are listed in Table 1. The main secondary metabolites identified were flavonoid glycosides, the main derivatives of quercetin and myricetin. The HPLC-PAD analysis also revealed the presence of peaks corresponding to the typical UV spectra of phenolic acid derivatives. In addition, a fukugentin derivative and flavanone glucosides were detected. The FIA-ESI-IT-MS technique is a powerful tool for the direct and rapid identification of constituents as it does not need any special sample processing. Therefore, it could be used as a starting method for the identification and authentication of *P. insignis* extracts for quality control and assurance.

**Figure 2:**
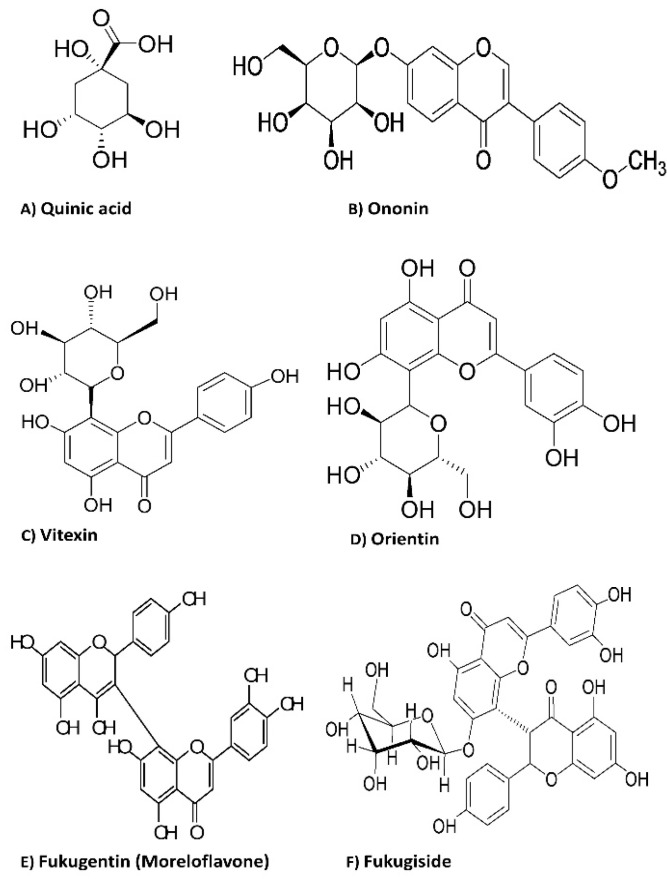
The chemical structures of the identified flavonoid glycosides detected in the crude ethanolic extract and fractions of *Platonia insignis*.

The chemical structures of the identified flavonoid glycosides are illustrated in Figure 2.

### 2.2. Antifungal Assays

#### 2.2.1. Determination of the Minimum Inhibitory Concentration (MIC) and Minimum Fungicidal Concentration (MFC)

The antifungal activity of PiHE and its fractions against *Candida* strains is listed in Table 2 and Table 3. For *C. albicans* isolates, PiHE MIC values ranged from 1.8 to 6.3 mg/mL, MFC from 3.6 to 14.6 mg/mL, and CFM/MIC from 1.3 to 3.6 mg/mL (Table 2). Comparatively, for *C. glabrata* isolates, PiHE MICs ranged from 1.6 to 8.3 mg/mL, MFCs from 3.1 to 16.7 mg/mL, and MFC/MIC from 1.0 to 3.3 mg/mL. DCMF showed MICs ranging from 0.7 to 3.1 and 1.0 to 20.8 mg/mL for *C. albicans* and *C. glabrata*, respectively, and MFCs from 2.6 to 10.4 and 1.8 to 20.8 mg/mL for *C. albicans* and *C. glabrata*, respectively (Table 2). The CFM/MIC ratio ranged from 2.9 to 6.4 for *C. albicans* and 3.6 to 41.7 for *C. glabrata*. EAF presented the best results in terms of the MIC values, ranging from 0.5 to 1.3 and 0.2 to 1.0 mg/mL for *C. albicans* and *C. glabrata*, respectively; CFM was between 0.5 and 2.6 and 0.3 and 1.6 mg/mL and the CFM/MIC ratio ranged between 0.8 and 2.0 and 1.5 and 2.5 for *C. albicans* and *C. glabrata*, respectively (Table 2).

Table 3 shows the geometric means of the MIC and MFC values of tested substances and the variation of these values for each isolate analyzed.

#### 2.2.2. Killing Assay

**Figure 3:**
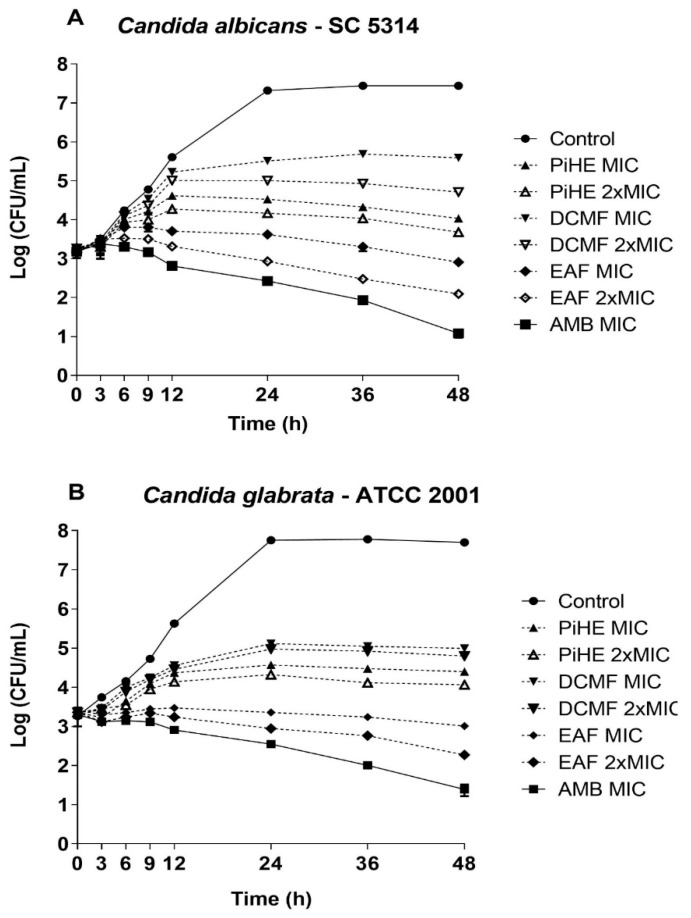
Time-kill curve for *C. albicans* SC 5314 (**A**) and *C. glabrata* ATCC 2001 (**B**) in the presence and absence of the *Platonia insignis* hydroethanolic extract and fractions. After different treatment times (0, 3, 6, 9, 12, 24, 36, and 48 h) at 37 °C for PiHE, DCMF, EAF (MIC and 2 × MIC), and AMB (MIC), 20 μL samples were withdrawn and subjected to serial dilutions before seeding on the Sabouraud Dextrose Agar (SDA) for counting colony-forming units (CFU)/mL. Data are presented as means of three biological replicates ± standard deviation and were analyzed at each time in comparison to the control samples. Symbols represent the quantification of colony-forming units grown at each tested time evaluated for each treatment.

The time-kill curve assay showed that both the extract and fractions of *P. insignis* significantly reduced the cellular growth of *C. albicans* SC 5314 within the first few hours of treatment (Figure 3A). PiHE at MIC, EAF at MIC, and 2 × MIC inhibited more than 50% growth of *Candida* spp. after 6 h of treatment and maintained the reduced cell counts compared to the untreated control, ultimately resulting in a growth reduction greater than 3 log 10 (99.9%) after 12 h of treatment, exhibiting a potential fungicidal action. DCMF had an inhibitory effect on *C. albicans* growth after 24 h of treatment compared to the control, with more than a 90% reduction rate. In the case of *C. glabrata* (Figure 3B), the PiHE showed growth reduction greater than 69% at 6 h of treatment and more than 90% inhibition compared to the untreated control at 12 h of treatment, reaching 99.9% (3 log 10) inhibition after 24 h of treatment. EAF showed a significant inhibitory action (*p* < 0.05), being able to reduce the growth of *C. glabrata* by more than 84% after 6 h of treatment and more than 90% in 9 h of treatment, and reached 99.9% (3 log 10) inhibition after 12 h of treatment. DCMF only reduced the growth of *C. glabrata* at 24 h of treatment, reaching about a 90% reduction; however, it was not able to totally eliminate the yeast cells. In conclusion, EAF showed stronger antifungal activity than PiHE and DCMF.

### 2.3. Antivirulence Activities

#### 2.3.1. Effect of PiHE, DCMF, and EAF on Cell Adhesion

**Figure 4:**
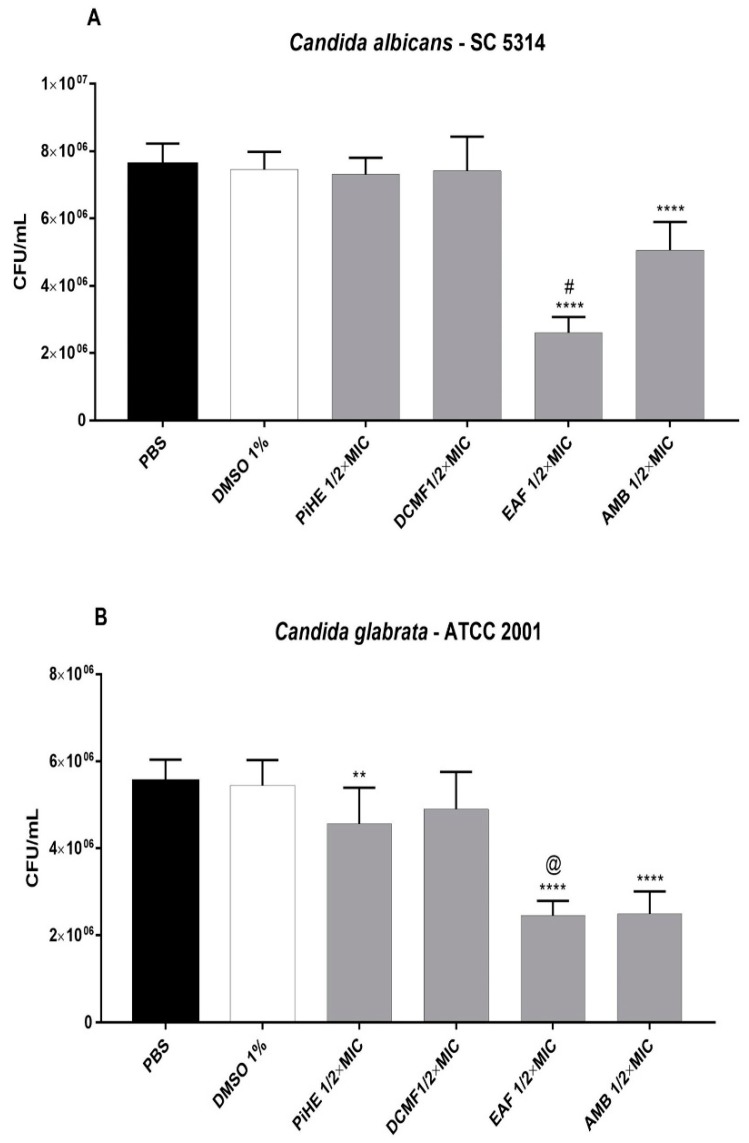
Effect of the hydroethanolic extract and fractions of *P. insignis* on the adhesion property of *C. albicans* (**A**) and *C. glabrata* (**B**). Cells were treated for 1.5 h and adhesion was analyzed for the reference strains in terms of CFU/mL. PBS, inoculum diluent (1 × 10^7^ cells/mL), and 1% DMSO were used as controls. Data were analyzed in relation to the PBS control by an analysis of variance with Tukey’s post-test, considering *p* < 0.05, and are represented as the mean ± standard deviation. (**) *p* < 0.01; (****) *p* < 0.0001; (#) *p* < 0.0001 with respect to PiHE, DCMF, and AMB; (@) *p* < 0.0001 with respect to PIHE and DCMF. AMB, amphotericin B; FLZ, fluconazole; PiHE, hydroethanolic extract of *P. insignis*; DCMF, dichloromethane fraction; EAF, ethyl acetate fraction. The tests were performed in quadruplicate.

The cell adhesion of *C. albicans* SC 531 cells was inhibited by EAF at ½ × MIC (*p* < 0.0001, Figure 4A). Both PiHE and EAF inhibited *C. glabrata* adhesion at ½ × MIC concentration (Figure 4B). EAF showed better anti-adherence activity compared to PiHE against both the tested strains (*p* < 0.0001; Figure 4). The anti-adherence activity of EAF was even better than that of AMB against *C. albicans* (*p* < 0.0001; Figure 4A).

#### 2.3.2. Effects of PiHE, DCMF, and EAF on Biofilm Formation and Pre-Formed Biofilms

**Figure 5:**
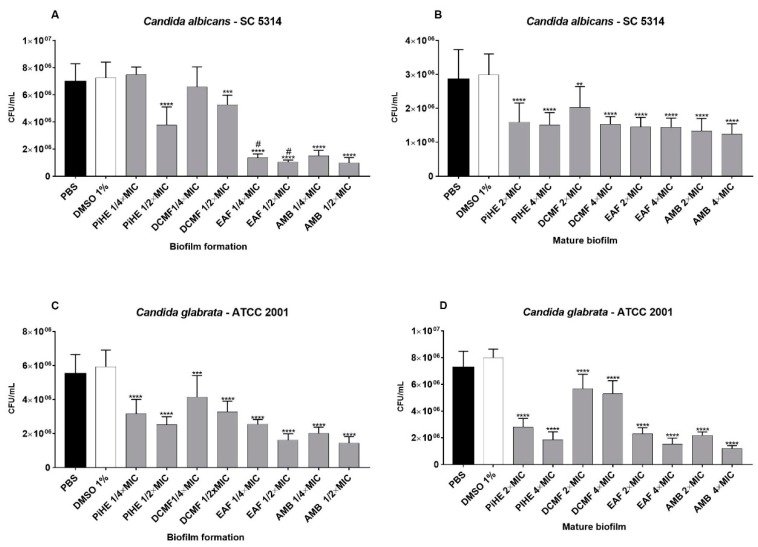
Effect of the *P. insignis* extract and fractions on *C. albicans* SC 5314 and *C. glabrata* ATCC 2001 biofilms. Biofilm formation and mature biofilms are expressed as CFU/mL (**A**–**D**). Results were compared to the vehicle control group (1% DMSO), positive control group (amphotericin ½ MIC, ¼ MIC, 2 × MIC, 4 × MIC) and negative control (PBS). Data are represented as the means of biological triplicates ± standard deviation and were analyzed by ANOVA, followed by Tukey’s post-test, with the significance of *p* < 0.05.

**Figure 6:**
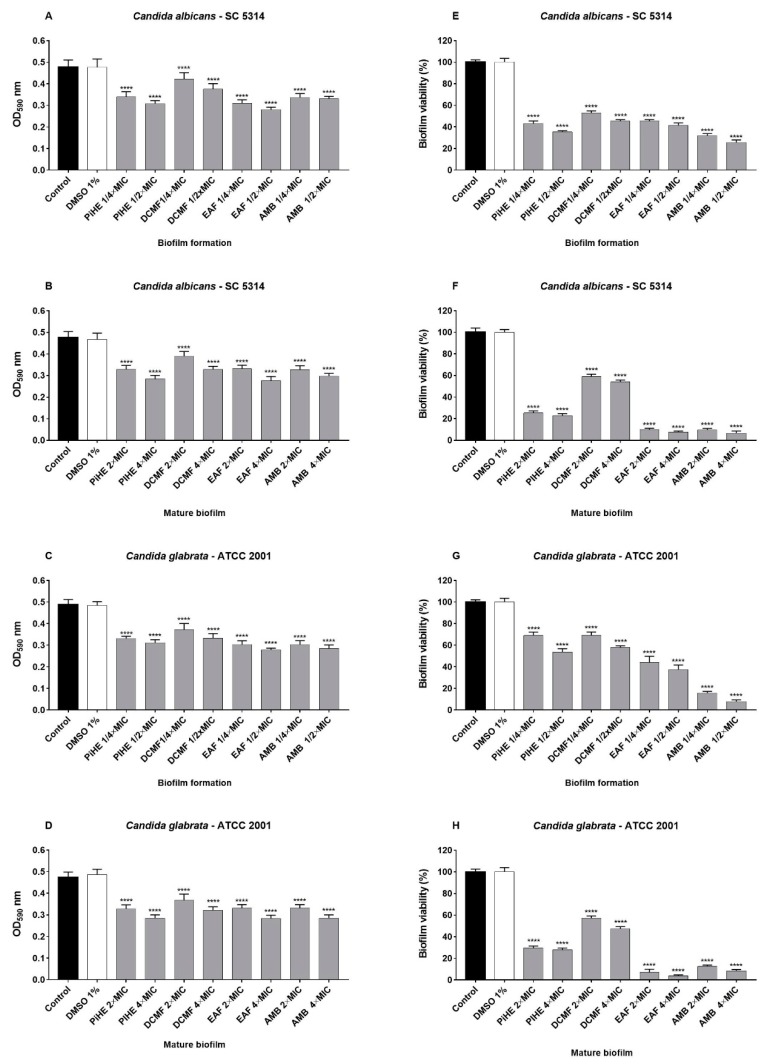
Effect of the *P. insignis* extract and fractions on *C. albicans* SC 5314 and *C. glabrata* ATCC 2001 biofilm formation and mature biofilm inhibition. Biofilm formation and mature biofilms were analyzed by measuring the absorbance at 590 nm (**A**–**D**) and percent viability (**E**–**H**). Metabolic activity was evaluated by the MTT reduction assay. Results were compared to the vehicle control group (1% DMSO), positive control group (amphotericin ½ MIC, ¼ MIC, 2 × MIC, 4 × MIC), and negative control (PBS). Data are represented as the mean ± standard deviation for three independent experiments with a significance of *p* < 0.05, and were analyzed by ANOVA, followed by Tukey’s post-test. * *p* < 0.05.

PiHE, DCMF, and EAF showed anti-biofilm activity on biofilm development, as well as on mature biofilms (Figure 5 and Figure 6). PiHE, DCMF, and EAF significantly reduced (*p* < 0.05) biofilm formation for *C. albicans* and *C. glabrata* and also had an inhibitory effect on pre-formed biofilms, compared to the control. EAF reduced *C. albicans* biofilm formation more than DCMF or PiHE (Figure 5A). PiHE and EAF were more effective in achieving the mature biofilm disruption of *C. glabrata* (Figure 5D). The anti-biofilm activity of the extracts was confirmed by biomass reduction analyses (Figure 6A–D) and by a metabolic activity assay (Figure 6E–H). EAF was highly potent in decreasing the viability (reduction >90%) of the mature biofilm for both the reference strains. 

### 2.4. Evaluation of Mitochondrial Membrane Potential by Flow Cytometry

**Figure 7:**
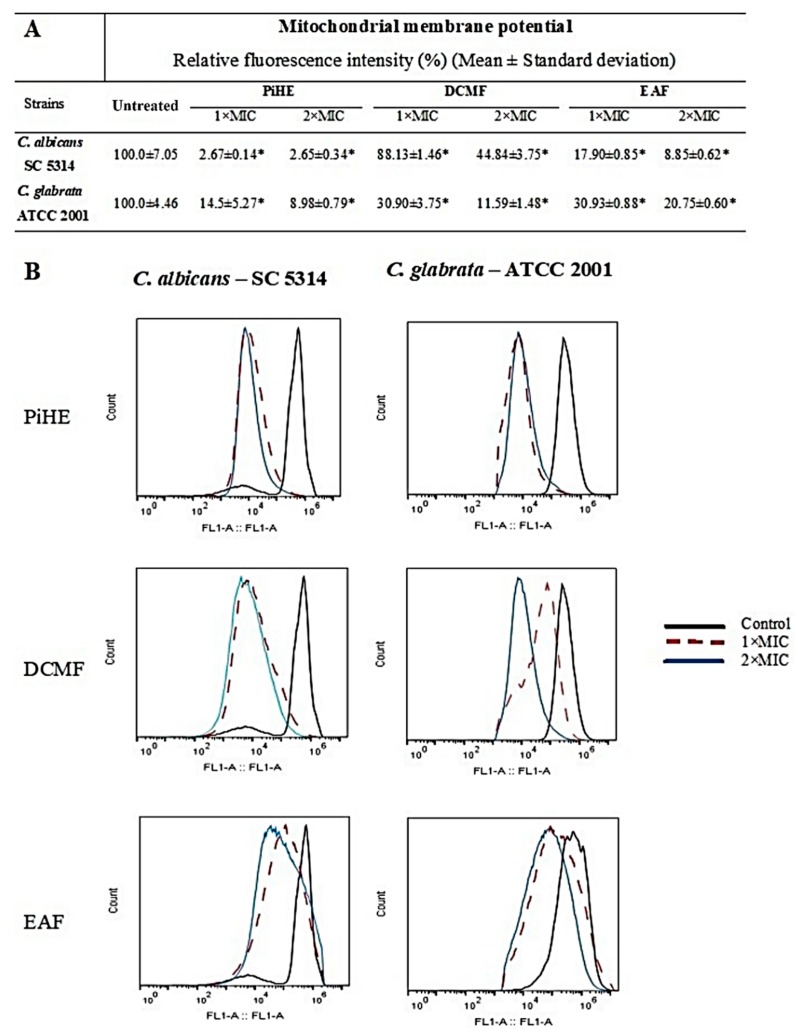
The *Platonia insignis* extract and fractions reduced the mitochondrial integrity of *C. albicans* and *C. glabrata* cells after 24 h of treatment. Data are shown as relative fluorescence intensity (**A**) and histogram (**B**) plots. Data were analyzed by ANOVA, followed by Tukey’s post-test, and statistical differences are represented by an asterisk, * *p* < 0.05. MIC, minimum inhibitory concentration; PiHE, hydroethanolic extract of *P. insignis*; DCMF, dichloromethane fraction; and EAF, ethyl acetate fraction.

The effect of PiHE, DCMF, and EAF on the mitochondrial membrane potential of *C. albicans* and *C. glabrata* cells was analyzed by flow cytometry. Untreated cells showed high fluorescence emission intensities due to the incorporation of Rhodamine 123 (Rho 123), whereas, upon treatment with the extracts, the fluorescence intensity was significantly reduced (*p* < 0.05). The reduction in the fluorescence intensity had a positive variation index (VI), indicating the depolarization of the mitochondrial membrane of *C. albicans* and *C. glabrata* (Figure 7). The relative fluorescence intensity in *C. albicans* cells was reduced to 2.67% and 2.65% (MIC and 2 × MIC, respectively) in the presence of PiHE, 88.13% and 44.84% (MIC and 2 × MIC, respectively) in the presence of DCMF, and 17.90% and 8.85% (MIC and 2 × MIC, respectively) with EAF. 

For *C. glabrata*, a reduction of 14.85% (MIC) and 8.98% (2 × MIC) in the relative fluorescence intensities was seen when treated with PiHE, while in the presence of DCMF, it was 30.90% and 11.59% for MIC and 2 × MIC, respectively. Finally, when treated with EAF, the relative fluorescence intensity reductions were 30.93% and 20.75%, respectively (Figure 7).

### 2.5. Evaluation of Lysosomal Membrane Integrity upon Treatment with PiHE, DCMF, and EAF

**Figure 8:**
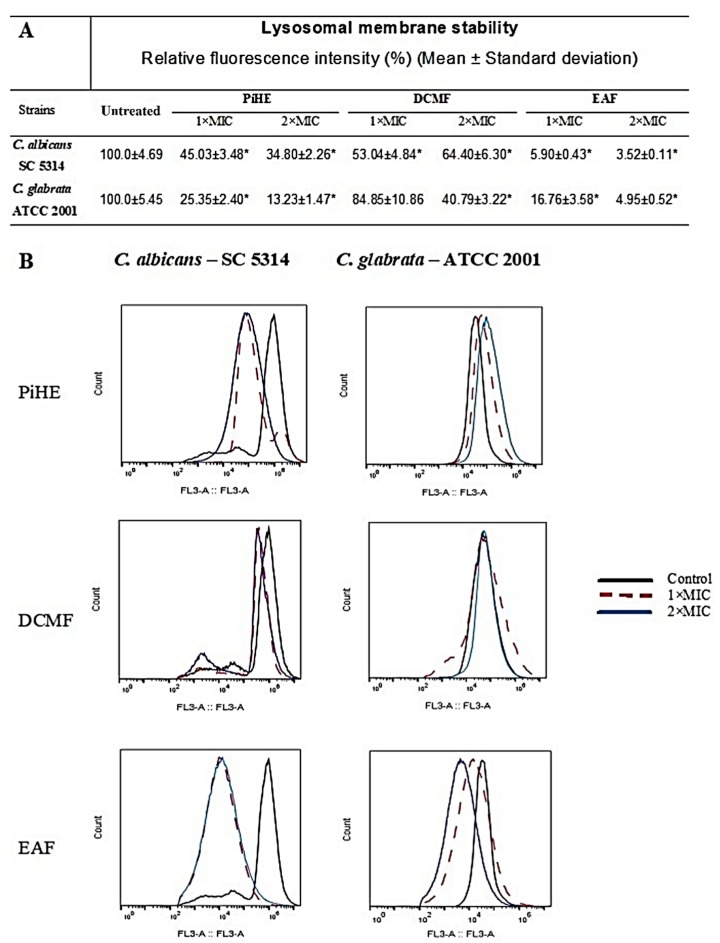
The *P. insignis* extract and fractions reduced the lysosomal integrity of *C. albicans* and *C. glabrata* cells after 24 h of treatment. Data are shown as the relative fluorescence intensity (**A**) and histograms (**B**). Data were analyzed by ANOVA, followed by Tukey’s post-test, and statistical differences are represented by an asterisk. * *p* < 0.05. MIC, minimum inhibitory concentration; PiHE, hydroethanolic extract of *P. insignis*; DCMF, dichloromethane fraction; and EAF, ethyl acetate fraction.

The effects of PiHE, DCMF, and EAF on the *Candida* cells were analyzed by the evaluation of the lysosomal membrane integrity. Untreated cells showed a strong fluorescence emission, confirming that these cells had intact lysosomes (Figure 8). Treatment with PiHE, DCMF, and EAF compromised the *Candida* cells’ lysosomal membrane, due to the observed reduction in the fluorescence intensity emitted when compared to the control group. Treatment with PiHE reduced the relative intensity in *C. albicans* cells to 45.03% (MIC) and 34.8% (2 × MIC) and in *C. glabrata* cells to 25.35% (MIC) and 13.23% (2 × MIC). DCMF reduced the fluorescence intensity in *C. albicans* to 53.04% (MIC) and 64.40% and to 40.79% (2 × MIC) in *C. glabrata*, respectively. EAF showed a reduction in the fluorescence intensity greater than 90% at both concentrations tested against *C. albicans*. In the case of *C. glabrata*, the reduction in the fluorescence intensity was between 83% and 95% (Figure 8).

### 2.6. Cellular Cytotoxicity Assay

**Figure 9:**
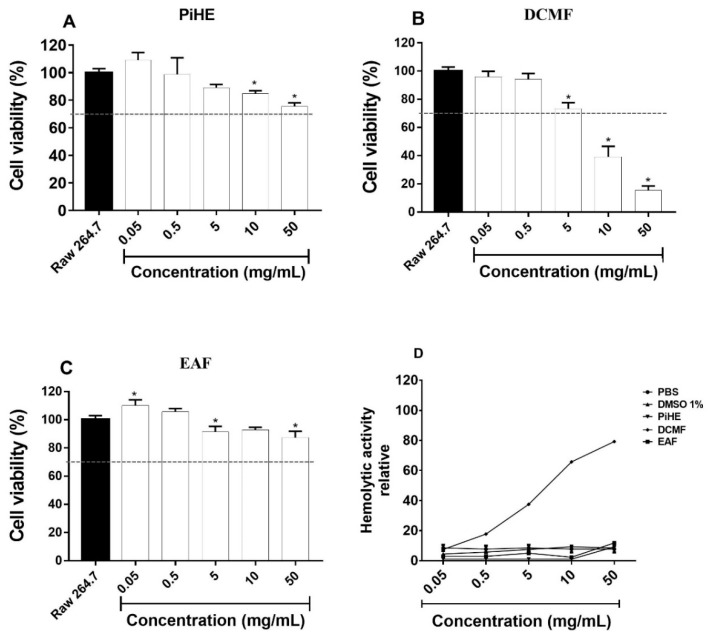
Effect of the *P. insignis* extract and fractions on RAW 264.7 cell viability (**A**–**C**) and on the hemolytic activity of sheep erythrocytes (**D**). Cells at the amount of 1 × 10^6^/mL were used in assays. The effects of PiHE, DCMF, and EAF on RAW 264.7 cells are shown, and viability above 70% was considered non-cytotoxic according to the ISO 10993-5 (2009) when compared to the untreated control (**A**–**C**). The percent hemolytic activity was compared to Triton X-100, as the hemolysis control (**D**). Triton X hemolytic activity was considered as 100% for data analysis. Data were analyzed by ANOVA with Tukey’s post-test, with * *p* < 0.05.

The cytotoxic effects of the *P. insignis* extract and fractions are shown in Figure 9. Even at high concentrations, PiHE and EAF did not show cellular toxicity on RAW 264.7 cells, (Figure 9A,C). PiHE and EAF also displayed low hemolytic activity (Figure 9D). The dichloromethane fraction (DCMF) exhibited dose-dependent cytotoxic activity as the viability was less than 50% at a 10 mg/mL concentration. DCMF also showed elevated hemolytic activity above a 5 mg/mL concentration (Figure 9B,D).

## 3. Discussion

With the increase in antibiotic-resistant *Candida* species, traditional antifungals are no longer effective in treating candidiasis. Therefore, it is highly necessary to identify new molecules that are non-toxic and more effective for developing therapies to treat *Candida* infections [8]. *Platonia insignis* (*bacurizeiro*) is an arboreal species native to the Brazilian Amazon and in particular, to the States of Maranhão and Piauí, where this species is found spontaneously. Its fruits are known for their pleasant taste and aroma and are highly appreciated by the Amazonian people [15]. Some studies have reported the biological activity of different extracts of *P. insignis* and the chemical components, such as Garcinielliptone FC, responsible for the biological activity, have already been isolated and identified [12,13,16]. However, the ethnopharmacological use of *P. insignis* is related to the use of the extracts of its seeds, bark, or fruit pulp because of its ability to heal, anti-inflammatory effects, leishmanicidal agents, antioxidants, and cytotoxic activities. This is the first study to report the antifungal activity of a hydroethanolic extract of *P. insignis* leaves and its fractions and thus is a pioneering investigation in the field of developing novel therapies to treat *Candida* infections.

In the present study, we evaluated the in vitro antifungal potential of a hydroethanolic extract of *P. insignis* leaves and the effects of the dichloromethane and ethyl acetate fractions against 16 clinical isolates of *Candida* spp. and reference strains *C. albicans* SC 5314 and *C. glabrata* ATCC 2001. Furthermore, we analyzed the complete phytochemical profile of its compounds by LC-ESI-IT-MS.

For a plant extract or fraction to have fungicidal potential, the MFC/MIC ratio must be less than 4 [17]. PiHE and its fractions showed an antifungal effect against the *Candida* isolates. PiHE had a fungicidal effect on isolates, with MICs ranging from 1.8 to 6.3 mg/mL against *C. albicans* and 1.6 to 8.3 mg/mL against *C. glabrata*. Among the tested fractions, EAF showed better antifungal activity, with a fungicidal effect on the isolates with relatively low MIC and MFC values. This anti-*Candida* action is mainly attributed to the presence of phytocompounds present in the extract and fractions evaluated. Fourteen compounds were identified by LC-ESI-IT-MS, among which were the glycosylated flavonoids derived from quercetin and myricetin, such as ononin and fukugentin. This chemical profile is completely new and different compared to those already described in the literature for extracts of *P. insignis* seeds that mainly comprise fatty acids, terpenes, xanthones, and phenolics as the major constituents. In addition, we found new compounds that have not been described as antifungal agents in large quantities in EAF, which explains the better antifungal activity of this fraction.

Though some pharmacological properties of *P. insignis*-derived compounds are known, their effects on *Candida* adhesion and biofilm formation, considered major concerns of the health care industry, have not been studied until now. PiHE and its fraction were able to inhibit the initial adhesion of *C. albicans* SC 5314 and *C. glabrata* ATCC 2001. EAF significantly inhibited the adhesion capacity of *Candida* strains at all tested concentrations and also at very low concentrations, especially in *C. glabrata* cells. These results are relevant since the first step in biofilm formation is the adhesion capacity, a process dependent on several adhesin proteins present in the *Candida* cells and which promotes the attachment to epithelia, other microorganisms, or abiotic substrates [18]. 

*Candida* spp. are known to form highly organized biofilms, especially in internal catheters and other prosthetic devices [19]. Furthermore, biofilms are known to be highly resistant to antifungals, which is a major hurdle in establishing an effective treatment [20,21]. Therefore, PiHE and its fraction are potential candidates for the development of effective antifungal drugs because they inhibited the formation of biofilms and significantly decreased the metabolic activity of preformed biofilms in the tested strains. We observed that the extract and fractions had a promising effect against preformed biofilms. Our results showed that there was a significant reduction in CFU/mL counts, and this was corroborated by crystal violet analysis (*p* < 0.0001) for both strains tested. These results were even more significant as EAF showed an inhibitory effect on the metabolic activity of the biofilms, exhibiting 90% inhibition for *C. albicans* and 96% inhibition for *C. glabrata*. These data suggest that the compounds have potent anti-biofilm activity and can be used as alternatives to anti-fungal drugs. *Candida* species have emerged as deadly human pathogens, particularly for immunocompromised individuals, because of the associated high mortality rates. Therefore, PiHE and EAF are promising new compounds for the development of drugs and phytotherapy with antifungal and anti-biofilm properties.

Surprisingly, extracts and fractions had a more pronounced effect on mature biofilm (48 h) than on biofilms in formation. The observation of this phenomenon may be due to the formation dynamics and the composition of biofilms in these different stages. In the early stages of biofilm formation, many planktonic cells are present, and the molecular structures and water content are different from those in a mature biofilm stage (48 h), which may have interfered with the mechanism of action and the efficiency of the extract and fractions of *P. insignis*. In this way, the extracts could be acting on extracellular matrix components, which are more present in mature biofilms. Similar results were obtained by Teodoro et al. [22] when studying the effects of a *Buchenavia tomentosa* acetone fraction on *Candida* biofilms.

Natural compound identification with anti-biofilm or anti-fungal activity against *Candida* is a difficult task. Recently, only a few therapeutic agents have demonstrated activities against fungal biofilms in vitro and/or in vivo [23]. Therefore, it is important to discover new anti-biofilm molecules. A previous study characterized 35 pharmacologically active compounds and verified that only one had an effect on *Candida albicans* biofilms and was not cytotoxic [24]. Alalwan et al. [25] performed different experiments with curcumin and found that this substance interferes with the adhesion of *C. albicans* SC5314 to denture materials. Teodoro et al. [22] demonstrated that an acetone fraction from a *Buchenavia tomentosa* extract had the ability to inhibit *C. albicans* ATCC 18804 and *C. albicans* SC 5314 adherence and to disrupt 48 h biofilm. However, they also verified that the acetone fraction and gallic acid showed slight to moderate toxicity to Vero cells.

This is the first report showing potent anti-*Candida* and anti-virulence activity for PiHE and EAF, and in addition, both of them were non-toxic towards keratinocytes, which makes PiHE and EAF ideal candidates for the treatment of candidiasis. When treated with higher concentrations of PiHE, RAW 264.7 cells had more than 70% viability, while EAF was not cytotoxic at any concentration tested. PiHE and EAF do not cause hemolysis, which also constitutes a fundamental characteristic of an ideal anti-fungal drug.

Furthermore, we decided to verify the action of the extract and fractions on fungal cells. The extracts triggered a perturbation of the mitochondrial membrane, which caused membrane depolarization. Therefore, the treatment of yeast cells with extracts altered the respiratory functions of the mitochondrial membrane and prevented Rho 123 from accumulating in the mitochondrial membrane [26]. The alteration of ΔΨm can cause a collapse of the membrane and would lead to a transient opening of pores in the membrane, releasing pro-apoptotic factors in the cytosol [27,28]. This, in part, provides a possible explanation for the antifungal potential of the extracts in relation to the number of flavonoid derivatives present in them. These also exhibited the presence of pro-oxidant factors [29], increasing intracellular free radical levels and causing damage in the mitochondrial membrane [27].

We observed that PiHE, DCMF, and EAF also damaged the lysosomal membrane in fungal cells. Lysosomes are acidic organelles that contain hydrolytic enzymes, which are involved in various cellular processes, such as post-translational protein maturation, receptor degradation, and the extracellular release of active enzymes [30]. Lysosomal membrane rupture causes release into the cytosol of cathepsin D and cysteine, which are classes of lysosomal proteases directly responsible for the induction of apoptosis [30]. The *P. insignis* extract and fractions caused the rupture of the lysosomal membrane of the treated cells, leading to a loss of lysosomal pH and the subsequent leakage of Acridine Orange (AO) in the cytosol [31], causing an increase in green fluorescence when compared to the untreated cells.

The *P. insignis* extract and fractions were not cytotoxic, so we think that their constituents probably interact with a specific component of the fungal membrane, such as ergosterol, or with one of the intermediates from the complex cell membrane synthesis pathway, which, in any case, would lead to a reduction of ergosterol. This, in turn, would lead to the disruption or opening of pores in the plasma membrane, resulting in the death of fungal cells.

Given the results obtained, in vitro, bioinformatics, and murine VVC model studies are underway to confirm the antifungal activity of the compounds found in EAF.

## 4. Materials and Methods 

### 4.1. Plant Material: Collection, Identification, and Extraction

Plant material (leaves) was collected between May and June 2017 in São Luís, Maranhão, Brazil (W44°13′35.1″ S2°31′38.2″). The species were identified and cataloged at Maranhão Herbarium, Center of Biological and Health Sciences from the Federal University of Maranhão, under the voucher specimen number 9722 identified by Prof. Eduardo B. de Almeida Jr. Extracts were prepared in the Chemistry Laboratory of Ceuma University. The leaves were sanitized and dried at 28 °C for seven days and then pulverized in a slicer (Te-651/2, Tecnal^®^, Piracicaba, SP, Brazil). Then, 200 g of plant material was 1/6 (m/v) diluted in 70% ethanol and macerated for five days at 28 °C with occasional stirring, and solvent was exchanged every 24 h. The extract was filtered, and the supernatant was concentrated in a rotary evaporator under reduced pressure at 40 °C (Quimis^®^, Modelo BOD Q-315M). 

The hydroethanolic extract of *P. insignis* (PiHE) (98% yield) was lyophilized (Liotop^®^, model L101); packed in a sterile, hermetically-sealed amber glass bottle; and stored at −20 °C until further use. The extract was sequentially subjected to liquid-liquid partition with dichloromethane (DCM) (Dinamica, SP), followed by ethyl acetate (EAT) (Dinamica, SP). PiHE (10 g) was suspended in 100 mL of water:methanol solution (8:2), followed by four liquid-liquid extractions with DCM and then, with EAT, resulting in two fractions: a dichloromethane fraction (DCMF) and an ethyl acetate fraction (EAF). The obtained fractions were concentrated, lyophilized, and stored at −20 °C until further use. 

### 4.2. Chemical Analyses

#### 4.2.1. HPLC Fingerprint Analysis

Experiments for phytochemical fingerprint analysis were carried out using a Thermo Scientific^®^ LCQ Fleet mass spectrometer system (Thermo Fisher Scientific, Waltham, MA, USA). The chromatographic separation was performed on Kinetex^®^ C18 (2.1 × 100 mm, 100 A, and 5 μm). The mobile phases used were 0.1% formic acid in water (A) and acetonitrile + 0.1% formic acid (B) added to 0.1% formic acid in an exploratory gradient starting with 10% to 100% B in 20 min at a flow rate of 1.0 mL/min. The sample was injected into the mass spectrometer from the HPLC system, where the sample was analysed online by ESI-MS in negative mode with an associated UV detector. The column temperature was maintained at 25 °C. The flow rate was 1 mL/min, and the online UV spectrum was monitored at the wavelength of 254 nm. 

#### 4.2.2. ESI-MS/MS Analysis

The ESI-MS/MS analysis was carried out using a Fleet LCQ mass spectrometer from Thermo Scientific^®^ equipped with an ESI source operating in Auto-MSn mode to obtain fragmentation. The negative ionization mode was applied, and the optimized instrument settings were set as follows: The sample was ionized with an ESI source and fragmentations were obtained in multiple stages (MS^n^) in an ion trap (IT)-type interface. All the spectra were generated and analysed in negative mode. The experimental conditions were as follows: capillary voltage, −35 V; spray voltage, −5000 V; capillary temperature, 350 °C; carrier gas, N_2_; and flow, 60 (arbitrary units). The track acquisition was set at *m/z* 100–2000, with two or more sweep events performed simultaneously in the spectrum. All data acquisition and analysis were performed using the Thermo Xcalibur ChemStation (Thermo Fisher Scientific).

### 4.3. Antifungal Assays

#### 4.3.1. Candida Strains, Growth Conditions, and Inoculum Preparation

Sixteen clinical vaginal isolates of *Candida* spp. were used in this study. Out of 16, 10 were *Candida albicans* isolates and 6 were *Candida glabrata* isolates. Two reference strains from the American Type Culture Collection (ATCC), *C. albicans* ATCC 90028 and *C. glabrata* ATCC 2001, and one *Candida albicans* wild-type strain from the Spain Collection (SC5314) were included as controls in the experiments. Clinical vaginal isolates were isolated in accordance with the Declaration of Helsinki, and the protocol was approved by the University Ceuma Ethics Committee (CEP/UNICEUMA N: 813.402/2014). Isolates were deposited in the collection of the Applied Microbiology Laboratory of Ceuma University São Luís-Ma, Brazil. All subjects gave their informed consent for inclusion before they participated in the research. All *Candida* isolates were identified by multiplex PCR. Reference strains were kindly donated by the São Paulo State University, Araraquara Dental School, São Paulo, Brazil.

For experiments, isolates were reactivated in Sabouraud Dextrose Agar (SDA; Kasvi, Italy) for 24 h at 37 °C. The inoculum was prepared in NaCl solution (0.85%) from 24 h-grown colonies and adjusted spectrophotometrically (Global Trade Technology) at a wavelength of 530 nm for a cell density equivalent to McFarland scale 0.5. For microdilution tests, the suspension was diluted in RPMI 1640, with glutamine, without bicarbonate (Sigma-Aldrich, St. Louis, MO, USA), pH 7.0, and buffered with MOPS (Morpholinepropanesulfonic acid, Sigma Chemical, St. Louis, MO, USA) at the concentration of 1 × 10^3^ to 5 × 10^3^ CFU/mL [32].

#### 4.3.2. Determination of the Minimum Inhibitory Concentration (MIC)

The minimal inhibitory concentration (MIC) was determined by a microdilution test [33]. Fluconazole (FLZ) and Amphotericin B (AMB) (Sigma-Aldrich, São Paulo, Brazil) were used as controls. PiHE and its fractions (200 mg/mL) were dissolved in 20% DMSO (Dimethylsulfoxide, Merck) and then diluted in RPMI-1640-MOPS to 0.024–100 mg/mL. FLZ was diluted in RPMI-MOPS to 0.125–256 µg/mL and AMB to 0.0313–16 µg/mL. RPMI-1640, without the antifungal extract, was used as the negative control in growth assays and 20% DMSO (v/v) was used as the vehicle control. Inoculum (100 µL) plus extracts or antifungals (100 µL) were incubated for 48 h at 35 °C. Results were analyzed visually and in a microplate reader at a 540 nm wavelength (Thermo Plate) [33]. MIC was defined as the lowest concentration of extract, fractions, FLZ, and AMB at which no visible growth was detected. In all experiments, the absorbance values obtained from wells containing extracts, fractions, or antifungals plus medium and inoculum were subtracted from those obtained from wells containing only extract or fractions plus medium. The percent growth inhibition for *Candida* spp. was determined using the formula described below:

(1) $$\%\;\mathrm{Inhibition}=\frac{Ac-At}{Ac}\;\times \;100$$

where *Ac* is the control absorbance and *At* is the test absorbance. All the tests were performed as biological triplicates.

#### 4.3.3. Determination of the Minimum Fungicide Concentration (MFC)

To determine the MFC value, 10 μL aliquots from the microplate wells that had concentrations above the MIC were sub-cultivated on SDA Petri dishes and incubated at 37 °C, for 24–48 h. MFC was defined as the lowest concentration of the extract which showed no colonies on the culture medium surface [32]. The MFC/MIC ratio was calculated to determine if substances had fungistatic (MFC/MIC > 4) or fungicidal (MFC/MIC < 4) action [17,34]. MFC assays were performed in biological triplicates. 

#### 4.3.4. Time-Kill Assay

*C. albicans* SC 5314 and *C. glabrata* ATCC 2001 standard strains were used to establish the time-kill curve. Assays were performed according to the previously published protocol in 96 well-plates, with some modifications [35]. The standard inoculum was diluted in RPMI 1640 to a final concentration of 5 × 10^3^ CFU/mL and added to the extracts distributed in 96-well plates. The extracts were used at concentrations of 2 × MIC and 4 × MIC. 1% DMSO (control vehicle), AMB (MIC; positive control) plus inoculum, and RPMI 1640 plus inoculum (negative control) were included in the tests as controls. Microplates were incubated at 37 °C. At different time points (0, 3, 6, 9, 24, 36, and 48 h), 20 μL aliquots were taken from each well and transferred to 96-well plates containing 180 µL of PBS to perform ten-fold serial dilutions for each sample. Then, a 10 μL aliquot from each dilution was plated on SDA and incubated at 37 °C for 48 h to determine the colony-forming units (CFU/mL). The tests were performed in biological quadruplets.

### 4.4. Antivirulence Activity

#### 4.4.1. Effect of PiHE and Fractions on Candida spp. Adhesion 

The assays were performed according to the previously published protocol, with some modifications [36]. Strains were grown in Yeast Nitrogen Base (YNB) plus glucose (50 mM) for 18 h at 37 °C. Samples were then centrifuged at 2060× *g* for 5 min, washed 2× with PBS, and suspended in PBS (5 mL), and their optical density was adjusted to 1 × 10^7^ CFU/mL. Inoculum (100 µL) plus extracts (100 µL. ½ MIC) were transferred to 96-well microplates and incubated for 90 min at 37 °C. Inoculum without extracts was used as a negative control. The supernatants were then discarded and the wells were washed twice with PBS. Subsequently, the adherent cells were resuspended in 100 μL of PBS. Samples were serially diluted to determine the CFU/mL after growth in SDA (48 h, 37 °C). The tests were performed in biological quadruplicate.

#### 4.4.2. Effect of Extract and Fractions on Biofilm

The assays were performed according to the previously published protocol [19], with certain modifications. The inoculum was cultured in Yeast Nitrogen Base Medium (YNB; Sigma-Aldrich) supplemented with 50 mM of glucose and was incubated at 37 °C for 18 h. The microbial suspension was then centrifuged at 1666× *g* (Centrifuge 5810R, Eppendorf) for five minutes, and the cell pellet was washed twice with PBS and then resuspended in 5 mL PBS. Inoculum optical densities were adjusted to 1 × 10^7^ CFU/mL. Furthermore, 200 μL from each of the yeast suspensions was transferred to 96-well polystyrene plates (Kasvi, Ortygia) and incubated for 90 min at 37 °C to achieve adhesion. Then supernatants were aspirated, and the wells were washed twice with sterile PBS to remove any non-adhered cells. 

To evaluate the inhibitory effects of the extracts on the formation of biofilms, 200 μL of each extract and fractions at sub-inhibitory concentrations of ¼ × MIC and ½ × MIC were diluted in YNB supplemented with 50 mM glucose and were added to each well with adherent cells, followed by incubation for 24 h 37 °C. After incubation, the supernatant was aspirated, biofilms formed were washed twice with PBS and then evaluated by counting the CFU/mL, and metabolic activity was determined by an MTT (3-methyl-[4-5-dimethylthiazol-2-yl]-2,5-diphenyltetrazolium) (Sigma-Aldrich, St. Louis, MO, USA) assay and by staining with crystal violet.

To analyze the effect of the extract and fractions on pre-formed biofilms, microplates with adherent cells were incubated for 48 h at 37 °C. Culturing media was exchanged after 24 h. Supernatants were aspirated, biofilms were washed twice with PBS, and extracts (200 μL; at 2 × MIC and 4 × MIC) diluted in YNB supplemented with 50 mM glucose were added to each of the wells. Biofilms were incubated for another 24 h at 37 °C. After incubation, the supernatants were aspirated, biofilms were washed twice with PBS and then analyzed for CFU/mL, and metabolic activity was determined by MTT and by staining with crystal violet. In all experiments, biofilms without extracts and biofilms with AMB were used as a growth control and positive control, respectively.

#### Colony Count Analysis of Biofilms 

After washing the biofilms twice with PBS, adhered biofilms were collected by scraping the bottom of each well plate and were suspended in 100 µL of PBS. CFU/mL was determined by serially diluting each sample of biofilm, followed by plating 20 μL of the suspension (quadruplets) on SDA and then counting the colonies after incubation at 37 °C for 48 h [37].

#### Biofilm Viability Assay

The measurement of the metabolic activity of the cells in the biofilm was determined by the MTT method, according to [38], with some modifications. After washing biofilms 3 × with PBS, 100 μL of MTT (5 mg/mL; Sigma, USA) was added to each sample, which was then incubated for 4 h in the dark. The supernatants were then removed. Next, 100 μL of DMSO was added to each well and the samples were incubated further for ten minutes. The plate absorbance was read in a microplate reader (Softmax^®^ Pro) at 570 nm. The experiments were performed six times on three different occasions.

#### Biofilm Analysis by Crystal Violet Staining

Biofilms were quantified using the crystal violet staining method [37]. Briefly, after washing the biofilms with PBS, they were dried at room temperature and fixed by resuspending them in 200 μL of 95% (v/v) methanol, followed by 15 min of incubation. Furthermore, the methanol was removed, and the plates were dried for 20 min at room temperature. Next, 200 μL of crystal violet (1% v/v) was added to each well and the samples were incubated for five minutes with the stain. Plates were washed twice with PBS and 200 μL of acetic acid (33% v/v) was added to each well. To obtain absorbance values, 100 μL from each of the sample wells was transferred to a new 96-well microplate and read at 570 nm in the microplate reader. The experiments were repeated six times on three different occasions.

### 4.5. Flow Cytometry Analysis for Examining the Effect of the Extract and Fractions on the Mitochondrial Membrane Potential (ΔΨm) and Lysosomal Membrane Stability

The stability of the lysosomal membrane and the mitochondrial membrane potential (ΔΨm) were assessed by flow cytometry using the Acridine Orange (AO) and Rhodamine 123 (Rho 123) fluorescence probes, respectively [39,40]. The assays were carried out with *C. albicans* SC 5314 and *C. glabrata* ATCC 2001 strains, according to the procedure of Alves et al. [31], with some modifications.

The strains were resuspended in 500 μL of MOPS-buffered RPMI-1640 at a density of 1.0 × 10^6^ cells/mL. The extract and fractions were added to the cell suspensions at concentrations of 1 × MIC and 2 × MIC and incubated for 24 h at 37 °C. After incubation, the cells were centrifuged at 3800× *g*, for 10 min (Centrifuge 5810R, Eppendorf), washed thrice with PBS (pH 7.2), resuspended in 500 μL of PBS, and labeled with AO (1 μg/mL in the dark, 20 min) or Rho 123 (10 μg/mL in the dark, 10 min). After incubation with the labels, the cells were washed thrice with PBS, resuspended in PBS, and analyzed by flow cytometry (BD Accuri ™, United States, FL3 channel for AO and FL1 for Rho123). The variation index (VI), calculated by the formula (MT-MC)/MC (MC = mean fluorescence intensity of control cells; MT = mean fluorescence intensity of treatments), showed the changes in the fluorescence intensity of Rho 123. When the mitochondrial membrane is depolarized, the VI values are negative.

### 4.6. Cytotoxicity Analysis Using Murine RAW 264.7 Cells

Murine RAW 264.7 cells were procured from the Cell Bank of Rio de Janeiro-Paul Ehrlich Scientific-Technical Association (APABCAM-Rio de Janeiro, RJ, Brazil) and maintained at the Immunophysiology Laboratory (LIF) of the Federal University of Maranhão. Cells were cultivated in Dulbecco’s high glucose modified Eagle’s medium (DMEM) supplemented with 10% fetal bovine serum (Sigma-Aldrich) and penicillin-streptomycin (1%) antibiotic solution. Cells were incubated at 37 °C under 5% CO_2_ and humidity. The culture media was renewed every 48 h and the cells were cultured until they reached 70% confluency. Cells were scrapped with a cell scarper for further experiments.

For the assay, 1 × 10^5^ cells/mL (100 μL) were plated in 96-well plates and incubated at 37 °C, under 5% CO_2_ for 4 h, to achieve cell adhesion. Next, the supernatant was removed, and the cells were treated with PiHE, DCMF, and EAF (0.05–50 mg/mL) and incubated for 24 h at 37 °C, under 5% CO_2_. 1% DMSO (vehicle), untreated cells, and cell-free media were used as control groups. After incubation, the supernatant was removed, and the adhered cells were evaluated by the MTT assay as follows. RPMI medium containing 5 mg/mL was placed in each well, MTT was added, and plates were incubated for 4 h at 37 °C under 5% CO_2_ in the dark. Supernatants were discarded, and the formed crystals were dissolved in 100 μL SDS (10% dodecyl sodium sulfate) overnight. The optical density (OD) was read at 570 nm using a microplate reader (Softmax^®^ Pro), and the cellular viability was expressed as a percentage of viable cells in relation to the positive control and reported as the mean of three independent assays [41].

### 4.7. Hemolytic Activity

Defibrinated sheep’s blood (EB FARMA, Rio de Janeiro, RJ, BR) was used for this assay. The erythrocytes were isolated by centrifugation at 290× *g* (Centrifuge 5810R, Eppendorf) for 10 min at 4 °C. After removal of the plasma, erythrocytes were washed three times with PBS (pH 7.4) and soon after, were resuspended in the same buffer at 2% (v/v). To evaluate the hemolytic activity of extracts and fractions, 100 μL aliquots of the erythrocyte solution were added to flat bottom 96-well microplates at different concentrations of the extract and fractions (0.05 to 50 mg/mL). Total hemolysis was achieved with 1% Triton x-100 (Sigma-Aldrich), while PBS was used as a negative control. 1% DMSO (vehicle) was also used as a control. After incubation for 60 min at room temperature, the cells were centrifuged at 300× *g* for 10 min and the supernatant was used to measure the absorbance at 540 nm [42]. The relative hemolytic activity was expressed in relation to Triton X-100, calculated by the following formula:

(2) Relative hemolytic activity (%)=[(As−Ab)×100]/(Ac−Ab),

where (*Ab*) is the control absorbance (blank, without extract), (*As*) stands for absorbance in the presence of the extract, and (*Ac*) stands for absorbance in the presence of Triton X-100. Assays were performed as biological quadruplets.

### 4.8. Statistical Analysis

Statistical analysis was performed using GraphPad Prism 7.00 Inc. Software. Normality tests were performed, and data were analyzed by a univariate analysis of variance (ANOVA) followed by the Tukey test, with a significance level of *p* < 0.05.

## 5. Conclusions

A pure hydroalcoholic extract and fractions from *P. insignis* leaves were active against *Candida* spp., but were not cytotoxic to RAW 264.7 cells. The extract and fractions also showed high anti-adhesion and anti-biofilm activities. EAF stood out as the best candidate as it was highly efficient in killing the fungal cells and significantly inhibited the virulence properties of *Candida* spp., such as cell adhesion and biofilm formation. The main antifungal chemical components in the extract were the glycosylated flavonoids derived from quercetin and myricetin, which interfered with or were bound to specific components of the fungal membrane. Our work contributes to the development of a new and efficient treatment of candidiasis and further studies are being developed in our laboratory using bioinformatics and animal models to further validate our data, as well as to test the topical use of these compounds.

## Figures and Tables

**Table 1:** Characterization of the compounds by direct injection in the mass spectrometer.

Sample	Number	[M − H]^−^	RT	MS^n^ Fragments	Names of the Suggested Structures
PiHE	1	191	18.80	173, 111, 85	Quinic acid
	2	429	18.61	267	Ononin
	3	431	18.43	295, 269	Vitexin
	4	447	18.24	429, 419, 285, 151	Orientin
	5	555	17.42	429, 401, 295, 267	Fukugentin
	6	573	16.33	447, 420, 419, 403, 296, 269,268	NI
	7	717	14.31	591, 565, 555, 429, 403, 401, 295, 267	Fukugiside
	8	761	12.49	634, 608, 431	NI
	9	777	10.35	447	NI
	10	1111	9.53	571, 537, 509, 429	Fukugentin dimer
	11	1113	8.04	555, 429	Fukugentin dimer
	12	1129	6.14	1111, 1003, 877, 555, 429	Fukugentin dimer
	13	1131	5.40	555, 429	NI
	14	1275	1.39	718, 429	NI
DCMF	15	573	8.34	557, 431, 295, 269	NI
	16	555	9.12	431, 429, 295, 269	Fukugentin
	17	429	9.56	429, 403, 295	Ononin
	18	561	10.10	539, 429, 413, 387	NI
EAF	19	573	6.40	447, 419, 269	NI
	20	717	6.62	657, 635, 555, 429	Fukugiside
	21	429	6.69	295, 269	Ononin
	22	555	7.90	429	Fukugentin
	23	1134	13.34	623, 555, 429, 299, 225	NI

Legend: PiHE, *Platonia insignis* hydroalcoholic extract; DCMF, dichloromethane fraction; EAF, ethyl acetate fraction; NI, not identified.

**Table 2:** Antifungal activities of the hydroethanolic extract of *Platonia insignis* and fractions against vaginal isolates of the *Candida* spp. and reference strains. All the values are presented in mg/mL.

	*Platonia insignis*									Antifungals	
STRAINS	PiHE			DCMF			EAF			AMB	FLZ
Candida albicans	MIC	MFC	MFC/MIC	MIC	MFC	MFC/MIC	MIC	MFC	MFC/MIC	MIC	MIC
A1 *Ca*	2.60	4.20	1.60	1.00	5.20	5.00	0.70	0.80	1.20	0.001	0.008
A2 *Ca*	2.60	5.20	2.00	1.00	3.60	3.50	1.00	1.00	1.00	0.0005	0.008
A3 *Ca*	1.80	3.60	2.00	3.10	10.40	3.30	0.50	0.50	1.00	0.00025	0.004
A4 *Ca*	2.60	8.30	3.20	1.30	4.20	3.20	1.30	2.60	2.00	0.0005	0.016
A5 *Ca*	4.20	8.30	2.00	1.00	4.20	4.00	1.30	2.60	2.00	0.0005	0.016
A6 *Ca*	4.20	7.30	1.80	1.00	4.20	4.00	1.30	2.60	2.00	0.0005	0.016
A7 *Ca*	2.60	9.40	3.60	0.70	4.20	6.40	1.30	1.90	1.50	0.001	0.016
A8 *Ca*	2.60	5.20	2.00	0.90	2.60	2.90	1.00	2.10	2.00	0.0005	0.008
A9 *Ca*	6.30	8.30	1.30	2.30	10.40	4.40	1.00	1.80	0.80	0.00025	0.008
A10 *Ca*	6.30	14.60	2.30	1.80	5.20	2.90	1.30	1.30	1.00	0.0005	0.016
*Candida glabrata*											
B1 *Cg*	2.60	3.10	1.20	1.80	3.60	2.00	0.30	0.70	2.50	0.0005	0.004
B2 *Cg*	3.10	3.10	1.00	2.10	3.60	1.80	0.30	0.40	1.50	0.002	0.004
B3 *Cg*	1.60	5.20	3.30	2.60	10.40	4.00	0.20	0.30	1.70	0.002	0.008
B4 *Cg*	3.60	5.20	1.40	4.20	10.40	2.50	1.00	1.60	1.50	0.001	0.008
B5 *Cg*	8.30	16.70	2.00	4.20	12.50	3.00	0.30	0.70	2.00	0.0005	0.008
B6 *Cg*	5.20	10.40	2.00	20.80	41.70	2.00	0.20	0.40	2.10	0.002	0.008
SC 5314-C*a*	2.080	6.30	3.00	1.00	1.80	1.80	1.00	1.30	1.30	0.0005	0.016
ATCC 2001-C*g*	5.20	8.30	1.60	8.30	20.80	2.50	0.50	1.30	2.50	0.00025	0.016
ATCC 90028-*Ca*	5.20	10.40	2.00	10.40	20.80	2.00	0.40	1.60	4.00	0.001	0.008

Legends: Ca, *Candida albicans;* Cg, *Candida glabrata*; SC 5314 C*a*, reference strain; PiHE, a hydroethanolic extract of *Platonia insignis;* DCMF, dichloromethane fraction; EAF, ethyl acetate fraction; AMB, amphotericin; FLZ, fluconazole.

**Table 3:** MIC/MFC geometric means, MIC/MFC ranges, MIC_90_, and MFC of 16 clinical isolates of *Candida* spp. and reference strains for each tested substance.

Species (n of Isolates)	Antifungal Agent	MIC mg/mL			MFC mg/mL		
		Range	Geometric Means	MIC_90_	Range	Geometric Means	MFC
*Candida albicans* (10)	PiHE	1.8–6.30	3.18	3.58	3.60–14.60	6.45	7.44
	DCMF	0.7–3.10	1.20	1.41	2.60–10.40	4.78	5.42
	EAF	0.50–1.30	0.98	1.07	0.50–2.60	1.46	1.72
*Candida glabrata* (6)	PiHE	1.60–8.30	3.45	4.07	2.30–25.00	5.69	7.28
	DCMF	1.8–20.8	3.74	5.95	3.60–41.70	9.09	13.70
	EAF	0.2–1.00	0.30	0.38	0.30–2.60	0.54	0.68
SC 5314-Ca	PiHE	0.40–3.12	1.71	2.08	4.80–8.6	6.25	6.30
	DCMF	0.70–1.80	0.96	1.00	1.56–3.12	1.63	1.80
	EAF	0.78–1.56	0.96	1.00	0.78–2.00	1.21	1.30
ATCC 2001-Cg	PiHE	3.12–6.30	5.11	5.20	6.20–12.50	7.93	8.30
	DCMF	6.30–12.50	8.01	8.30	12.10–25.00	19.79	20.8
	EAF	0.39–0.78	0.49	0.50	0.78–2.20	1.19	1.30
ATCC 90028-Ca	PiHE	3.10–6.30	5.08	5.20	6.20–25.00	9.49	10.40
	DCMF	6.30–12.50	10.17	10.40	12.10–25.00	19.79	20.8
	EAF	0.10–1.00	0.37	0.40	0.90–2.30	1.52	1.60

Legends: Ca, *Candida albicans;* Cg, *Candida glabrata*; SC 5314 C*a*, wild-type reference strain; PiHE, *Platonia insignis* hydroethanolic extract*;* DCMF, dichloromethane fraction; EAF, ethyl acetate fraction; MIC, minimum inhibitory concentration; and MFC, minimum fungicide concentration.
